# Immunoprofiling of early, untreated rheumatoid arthritis using mass cytometry reveals an activated basophil subset inversely linked to ACPA status

**DOI:** 10.1186/s13075-021-02630-8

**Published:** 2021-10-29

**Authors:** H. Koppejan, M. Hameetman, G. Beyrend, V. van Unen, J. C. Kwekkeboom, A. H. van der Helm-van Mil, R. E. M. Toes, F. A. van Gaalen

**Affiliations:** 1 Department of Rheumatology, Leiden University Medical Center, PO box 9600 (Zone C1-R), Albinusdreef 2, 2233 ZA Leiden, The Netherlands; 2 Flow Core Facility, Leiden University Medical Center, Leiden, The Netherlands; 3 Department of Immunology, Leiden University Medical Center, Leiden, The Netherlands; 4 Institute for Immunity, Transplantation and Infection, Stanford University School of Medicine, Stanford, CA USA

**Keywords:** Rheumatoid arthritis, Mass cytometry, Immune-profiling, Basophils, CD62L

## Abstract

**Background:**

Autoantibody production is a hallmark of rheumatoid arthritis (RA). Anti-citrullinated protein antibodies (ACPA) are highly disease-specific, and their presence is associated with more severe disease and poor prognosis compared to ACPA-negative patients. However, the immune cell composition associated with antibody-positive/negative disease is incompletely defined. Mass cytometry (MC) is a high-dimensional technique offering new possibilities in the determination of the immune cell composition in rheumatic diseases. Here, we set up a broad phenotyping panel to study the immune cell profile of early untreated RA to investigate if specific immune cell subsets are associated with ACPA+ versus ACPA− RA.

**Methods:**

Freshly obtained PBMCs of early, untreated RA patients (8 ACPA+ and 7 ACPA−) were analysed using a 36-marker MC panel, including markers related to various immune lineages. Data were processed using Cytosplore for dimensional reduction (HSNE) and clustering. Groups were compared using Cytofast. A second validation cohort of cryopreserved PBMCs obtained from early RA patients (27 ACPA+ and 20 ACPA−) was used to confirm MC data by flow cytometry (FC). FC data were processed and analysed using both an unsupervised analysis pipeline and through manual gating.

**Results:**

MC indicated no differences when comparing major immune lineages (i.e. monocytes, T and B cells), but highlighted two innate subsets: CD62L^+^ basophils (*p* = 0.33) and a subset of CD16^−^ NK cells (*p* = 0.063). Although the NK cell subset did not replicate by FC, FC replication confirmed the difference in CD62L^+^ basophil frequency when comparing ACPA+ to ACPA− patients (mean 0.32% vs. 0.13%; *p* = 0.01).

**Conclusions:**

Although no differences in major lineages were found between early ACPA+ and ACPA− RA, this study identified the reduced presence of activated basophils in ACPA-negative disease as compared to ACPA-positive disease and thereby provides the first evidence for a connection between activated basophils and ACPA status.

**Supplementary Information:**

The online version contains supplementary material available at 10.1186/s13075-021-02630-8.

## Background

Rheumatoid arthritis (RA), a common chronic autoimmune disease, is characterized by persistent synovial and systemic inflammation, potentially leading to irreversible joint damage. A hallmark of RA is the presence of autoantibodies, such as rheumatoid factor (RF) and anti-citrullinated protein antibodies (ACPA). ACPA are highly disease-specific, and their presence is associated with more severe disease and poor prognosis compared to ACPA-negative patients (ACPA−) [1–3].

The pathogenesis of RA is incompletely understood, apart from a role for B cells, given the efficacy of B cell-depleting therapies [4, 5]. Despite great efforts by the field, data on the immune cell composition truly associated with RA is limited. Several studies investigating RA on the immune cell level report on decreased frequencies of regulatory T cells in RA patients [6]. Likewise, CCR6 expression was reported to be upregulated in CD4^+^CD45RO^+^ T helper cells in ACPA-positive (ACPA+) disease, as was an increase in the frequency of CD4, CD8 double-positive T cells and the ratio of M1/M2 monocytes in peripheral blood [7–9]. In contrast, a signature CD4^+^ T cells compatible with IL-6-mediated STAT3 signalling was observed mostly in ACPA− disease during the early clinical phase [10]. These studies generally focused on a specific cell type or immune cell subset present in the immune system providing limited information on the overall cellular composition of the major immune lineages in RA and/or RA endotypes. Moreover, few findings have been replicated which could be due to treatment effects or differences in disease duration.

The analyses of the immune cell composition by high-dimensional single-cell platforms such as mass cytometry (MC), also known as cytometry by time of flight, offer new possibilities to gain additional insights into the cellular composition within rheumatic diseases. MC is an antibody-based technique utilizing heavy metal isotope-conjugated probes [11]. Until now, MC has been used only to a limited extent to investigate rheumatic diseases. Nonetheless, the first studies using MC have revealed novel insights as suggesting, for example, that that PD-1^hi^CXCR5^−^CD4^+^ T cells (Tph cells) are expanded in joints and blood of seropositive RA [12]. Other studies using MC specifically reported differences in T and B cell, monocytes and neutrophils in RA as compared to controls but not comparing ACPA-positive and ACPA-negative disease [5, 13–15].

Given that in RA the production of ACPA antibodies is associated with a more severe clinical course, we hypothesized that differences at the immune cell level and in particular in T and B cell compartments are detectable between ACPA+ and ACPA− RA. Since MC allows the detailed profiling of the immune cell composition in RA, we now sought to probe the immune system in early, untreated RA, reducing possible confounding factors associated with treatment or symptom duration. Here, we describe an immune profile of early untreated RA to investigate if specific immune cells or subsets could be associated with the differences observed on a clinical level.

## Methods

### Patient material

For mass cytometry (MC) experiments, heparin blood was collected from untreated patients with polyarthritis of recent onset, who were enrolled into the Leiden Early Arthritis Clinic (EAC) cohort study (baseline EAC visit) [16]. All patients were diagnosed with RA and fulfilled the 2010 classification criteria for RA within the first year of follow-up (ACPA+ *n* = 8, ACPA− *n* = 7).

For flow cytometry (FC) experiments, peripheral blood mononuclear cells (PBMCs) were previously collected from RA patients enrolled in the EAC study and cryopreserved until use. A total of 47 patients diagnosed with RA (27 ACPA+ and 20 ACPA−) fulfilling the 1987 ACR criteria [17] were randomly selected. The EAC study was approved by the LUMC ethical committee, and all patients provided written informed consent. The patient’s characteristics are summarized in Table 1.

**Table 1:** Patient clinical characteristics of rheumatoid arthritis patients used in this study

	MC RA ACPA^+^	MC RA ACPA^−^	FC RA ACPA^+^	FC RA ACPA^−^
	*n* = 8	*n* = 7	*n* = 27	*n* = 20
Age in years, average (range)	64 (48–75)	67 (54–79)	55 (17–81)	51 (21–86)
Females, *n* (%)	4 (50)	4 (57)	16 (59)	12 (60)
RF positive, *n* (%)	8 (100)	2 (29)	19(79)	5(21)
Elevated CRP, *n* (%)	5 (50)^a^	3 (25)	62(24)	38(15)
SJC, mean (range)^b^	9 (3–20)	14 (4–29)	5 (0–20)	3 (0–16)
TJC, mean (range)^b^	11 (2–20)	17 (1–34)	3 (0–16)	3 (0–18)
DMARD usage, *n* (%)^c^	0 (0)	0(0)	6 (31%)	4 (36%)
Disease duration (in days), average (range)^d^	132 (13–600)	42 (21–60)	108 (5–542)	132 (3–610)

^a^No CRP was recorded for one male and one female (male ESR = 34, female ESR = 126)

^b^For 3 ACPA+ and 2 ACPA−, no SJC/TJC was recorded in the FC dataset

^c^For 17 RA patients, no DMARD usage was recorded in the FC dataset, frequency of DMARD calculation based on *n* = 19 in ACPA+ and *n* = 11 in ACPA−. DMARD usage included hydroxychloroquine, sulfasalazine and methotrexate

^d^For 1 ACPA+ and 1 ACPA−, no disease duration was recorded in the FC dataset

*MC* mass cytometry, *FC* flow cytometry, *ACPA* anti-citrullinated protein antibodies, *RF* rheumatoid factor, *CRP* C-reactive protein, *SJC* swollen joint count, *TJC* tender joint count, *DMARD* disease-modifying anti-rheumatic drugs

### Sample processing for mass cytometry

PBMC isolation from heparin blood was performed by Ficoll-Paque gradient centrifugation. Freshly isolated PBMCs (3 × 10^6^ cells) were directly stained with Cell-ID intercalator-103Rh to identify dead cells (Fluidigm, South San Francisco, CA, USA). Upon ^103^Rho staining, cells were fixed in freshly prepared 1.85% formaldehyde solution (Sigma-Aldrich, Darmstadt, Germany; diluted in Maxpar PBS, Fluidigm) and stored in Maxpar Cell staining buffer (Fluidigm) at 4C. Fixed samples were stored in suspension for a maximum of 3 days.

### Staining and acquisition for mass cytometry

All MC antibodies and their respective suppliers are summarized in Supplementary Table 1. Antibodies were either pre-conjugated to metals (Fluidigm) or in-house conjugated at 100-μg scale using a Maxpar X8 antibody labelling kit according to the manufacturer’s protocol (Fluidigm). All in-house conjugated antibodies were stored in 100μl antibody stabilization buffer supplemented with 0.05% sodium azide. All antibodies (pre- or in-house conjugated) were tested in a serial dilution staining to determine the optimal signal with minimal background. Staining was performed according to the manufacturer’s protocol (Fluidigm). Briefly, freshly fixed PBMCs were pre-incubated with Fc-block (Human TruStain FcX, Biolegend, San Diego, CA, USA), stained for 35 surface markers in the presence of Fc-block, permeabilized (eBioscience Foxp3/transcription factor staining buffer set, eBioscience, San Diego, CA, USA), and stained for intranuclear Ki-67, and finally, all cells are stained with Cell-ID intercalator-Iridium (^191^Ir and ^193^Ir, Fluidigm) diluted into Maxpar Fix and Perm Buffer (Fluidigm). Samples were stored as a pellet at 4 °C for a maximum of 3 days until MC acquisition. Prior to acquisition, samples were washed with Maxpar Cell Staining buffer and Maxpar Water (Fluidigm). Cells were diluted in Maxpar Water (Fluidigm) to approx. 0.75 × 10^6^ cells/ml and 10% v/v EQ beads were added (EQ Four Element Calibration Beads, Fluidigm). Samples were acquired on a Fluidigm Helios CyTOF system using HT injector, normalized using reference EQ passport P13H2302 within the Fluidigm acquisition software, and converted to FCS files. To monitor technical variation, a cryopreserved PBMC reference sample was included (buffy coat obtained from Sanquin, The Netherlands).

### Mass cytometry data analysis

FCS files were manually processed in FlowJo v10 (TreeStar, Ashland, OR, USA) to gate single, live, CD45^+^ cells and exported into new FCS files (Supplementary Figure 1). All processed samples combined resulted in a dataset of 6.4 × 10^6^ CD45^+^ cells originating from 15 different samples.

All CD45^+^ cells were sample-tagged, hyperbolic ArcSinh-5 transformed, and simultaneously subjected to dimension reduction in Cytosplore (v2.2) [18]. We used hierarchical stochastic neighbour embedding (HSNE) [19], preventing data loss linked to downsampling prior to dimension reduction and clustering methods. Major immune lineages were identified through the use of a 5-level HSNE taking all 36 markers into account, using default perplexity and iterations (30 and 1000, respectively). Individual lineages were further investigated within Cytosplore by ‘zoom in’ to a maximum of 0.3 × 10^6^ landmarks. Both overview and ‘zoomed’ levels were subjected to Gaussian mean shift (GMS) clustering within Cytosplore. Clusters were exported as new FCS files, including sample tags. Downstream analysis and comparisons were performed using Cytofast [20]. The generated marker expression heatmap and HSNE map acted as guidelines to determine the expression pattern for clusters of interest. The selected markers were used in a flow cytometry panel to perform an independent replication study on a second set of EAC samples as confirmation of the MC outcome.

### Flow cytometry

In a separate experiment, two cell subsets identified by MC were investigated using flow cytometry (FC). To this end, PBMCs of 47 untreated early RA patients (not previously used in MC) were thawed and stained with Zombie Yellow fixable Viability Kit (Biolegend). Samples were stained for surface markers resembling clusters of interest identified with MC, using BV605 as a general dump channel to exclude non-relevant cells. FC samples were used directly without the need of fixation. All FC antibodies and their respective suppliers are listed in Supplementary Table 2. As each antibody clone may have a slightly different epitope, we ensured that clones used in FC are similar to those used in MC. All samples were acquired and unmixed on a 3-laser Cytek^TM^ Aurora spectral cytometer (Cytek Biosciences Inc, Fremont, CA, USA). In contrast to conventional flow, spectral flow cytometry captures the full emission spectrum of every fluorescent molecule and allows unmixing based on the differences in the overall spectral signatures. FC samples were processed using FlowJo v10 for gating of single/live/CD45^+^ cells (Supplementary Figure 2). Pre-processed samples were either analysed using Cytosplore or manual gating in FlowJo. The ArcSinh cofactor needed for Cytosplore analysis was determined for each fluorochrome separately and applied to all samples as described by Melsen et al. (Github: https://github.com/janinemelsen/Single-cell-analysis-flow-cytometry) [21]. In case of manual analysis, pre-gated CD45^+^ samples were further gated in FlowJo v10 based on the expression pattern observed in the MC data.

Statistical analysis was performed in R using Cytofast when analysing >2 clusters simultaneously (MC data only) or using GraphPad Prism v8 (GraphPad Software, La Jolla, CA, USA). Statistical significance was considered when *p* < 0.05.

## Results

### Analyses of peripheral blood cells from early ACPA+ and ACPA− RA patients by a 36-marker MC panel

**Fig. 1:**
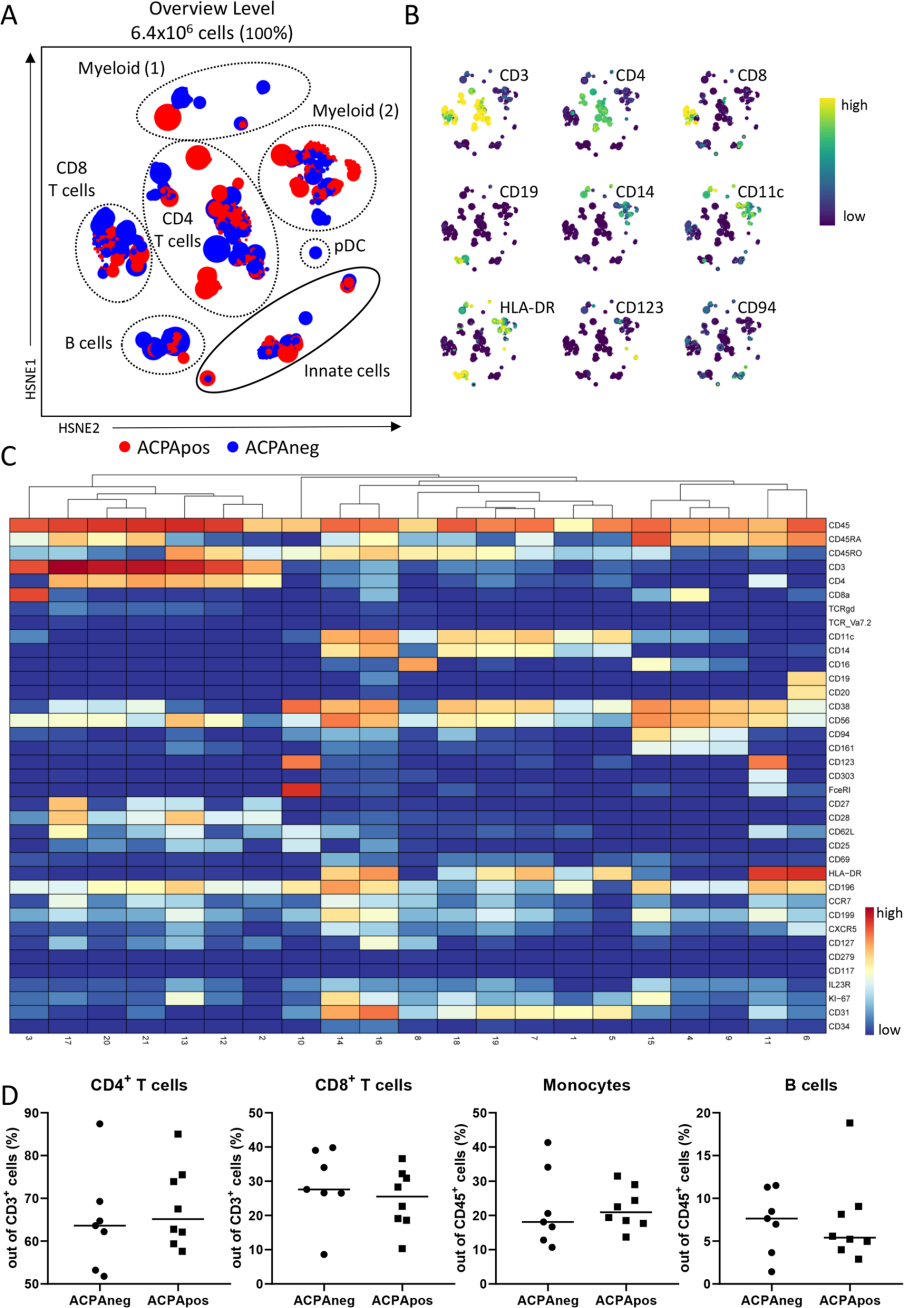
Analysis of major lineages revealed no differences between ACPA+ and ACPA− patients. **A** Five-level HSNE separated all major lineages as indicated by dashed circles, colour-coded for ACPA status (positive = red, negative = blue). The solid circle indicates innate landmarks. **B** Examples of expression patterns of the HSNE map shown in **A**. **C** Heatmap of clusters identified through default clustering in Cytosplore (Gaussian mean shift) showing the expression pattern. **D** Manual gating of major lineages showed no significant difference in frequency between ACPA+ and ACPA−

After applying the 36-marker mass cytometry (MC) panel to 15 untreated, early ACPA+ and ACPA− RA patients (Table 1), we first performed a 5-level HSNE dimension reduction on all 15 samples simultaneously, showing a clear separation of different major immune lineages such as T cells, B cells and monocytes (Fig. 1A, B, dashed lines). The HSNE [17] map also depicted landmarks representing the innate lineage (Fig. 1A, solid line). Default clustering in Cytosplore [22] resulted in 21 different clusters at the overview level (Fig. 1C). Analyses of these clusters by Cytofast [18] indicated that none of the cluster frequencies was significantly different between ACPA+ and ACPA− RA (Supplementary Figure 3). Although some of the ACPA− samples clustered together, this was not consistent for all ACPA-negative patients (Supplementary Figure 3A). Nonetheless, a trend towards the increased presence of CD27^low^CD28^low^CD45RA^+^CD4^+^ T cells (cluster 21) was observed in ACPA+ patients (3/8 patients), as this cluster was absent in all ACPA− patients (Supplementary Figure 3C). This cluster probably represents a subset of terminally differentiated effector memory T cells (T_EMRA_) based on their loss of CD27/CD28 combined with the expression of CD45RA.

In agreement with the results presented above, manual gating of the major immune lineages in FlowJo did not result in any significant differences (Fig. 1D), confirming that the overall composition of the major immune cell lineages in peripheral blood is similar between ACPA− and ACPA+ patients.

### HSNE of solely innate lineage identified two clusters: differences in basophil and NK cell subsets

Next, we investigated whether differences would become apparent when analysing subsets on a more detailed level. To this end, Cytosplore was used to zoom in on each lineage, analysing CD4 T cells, CD8 T cells, monocytes, B cells and the innate subsets in a separate analysis (zooming in to a maximum of 0.3 × 10^6^ landmarks in a single HSNE map). We did not identify any (significant) differences when analysing the major lineage subsets separately (data not shown).

**Fig. 2:**
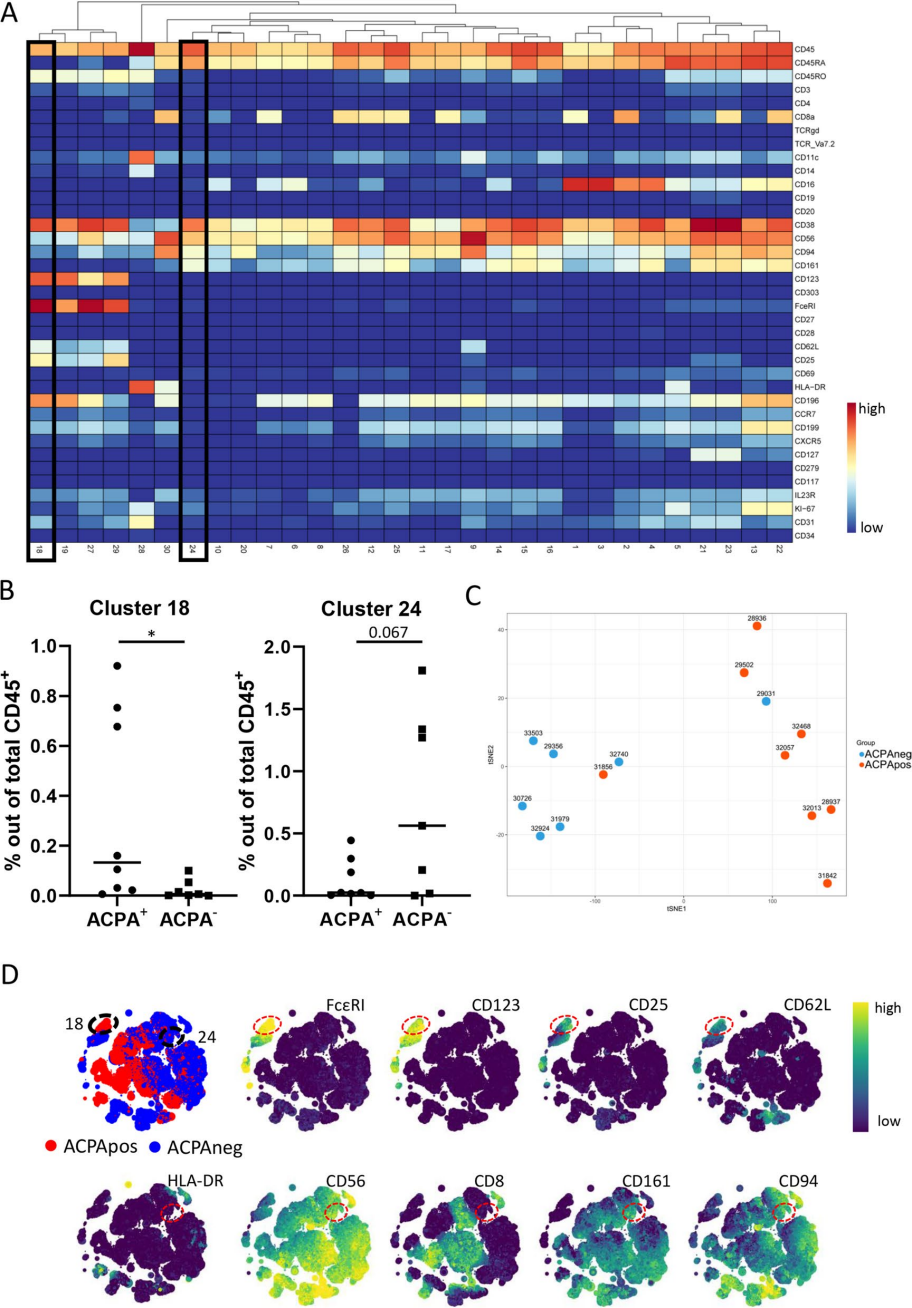
HSNE analysis of all innate subsets indicated differences in basophil and NK cell subsets. **A** Heatmap representing all clusters within the innate population and their respective expression patterns, cluster 18/24 indicated with black boxes. **B** Cluster 18 (basophil subset, *p* = 0.033) was reduced in ACPA− patients and there was a strong trend for cluster 24 (NK cell subset *p* = 0.063) present only in ACPA− patients (Cytofast). **C** tSNE plotting samples using clusters 18 and 24 as input confirmed the separation shown in **B**. **D** HSNE map indicating cluster 18 and cluster 24 (dashed circles) and examples of the expressed markers. ACPA+ in red, ACPA− in blue

All the innate subsets combined represented 14.5% of the total data set (0.93 × 10^6^ out of 6.4 × 10^6^ cells), and default clustering resulted in 30 clusters (Fig. 2A). To delineate the cluster frequency distribution for ACPA+ and ACPA− samples, we used Cytofast revealing that 2 clusters were differentially present in ACPA+ vs. ACPA− patients: clusters 18 and 24 (Fig. 2A—black boxes). The frequency of cluster 18 was significantly lower in ACPA− RA (*p* = 0.033), whereas the frequency of cluster 24 appeared higher in ACPA− RA (*p* = 0.063) (Fig. 2B). Furthermore, applying a dimension reduction based on cell cluster 18 and 24 frequencies indicated a clear separation of samples when colour-coded for ACPA status (Fig. 2C).

Cluster 18 expressed markers linked to basophils such as FcεRI, CD123, CD45RO, and additionally expressed CD25, CD62L and CCR6 (Fig. 2A, D). The remaining FcεRI-negative clusters appeared to be more similar in ACPA− and ACPA+ RA, though differences in expression can be observed, e.g. for CD8 or CCR6. Cluster 24 was lineage-negative (incl. HLA-DR and CD16) and expressed markers often observed on CD16^−^ NK cells or innate lymphocyte cells (ILCs) such as CD56, CD94 and CD161 (Fig. 2A, D). As no clear CD127 staining is observed, we indicate cluster 24 as a subset of NK cells.

### Flow cytometry replication—confirmation of CD62L^+^ basophils inversy linked to ACPA status

**Fig. 3:**
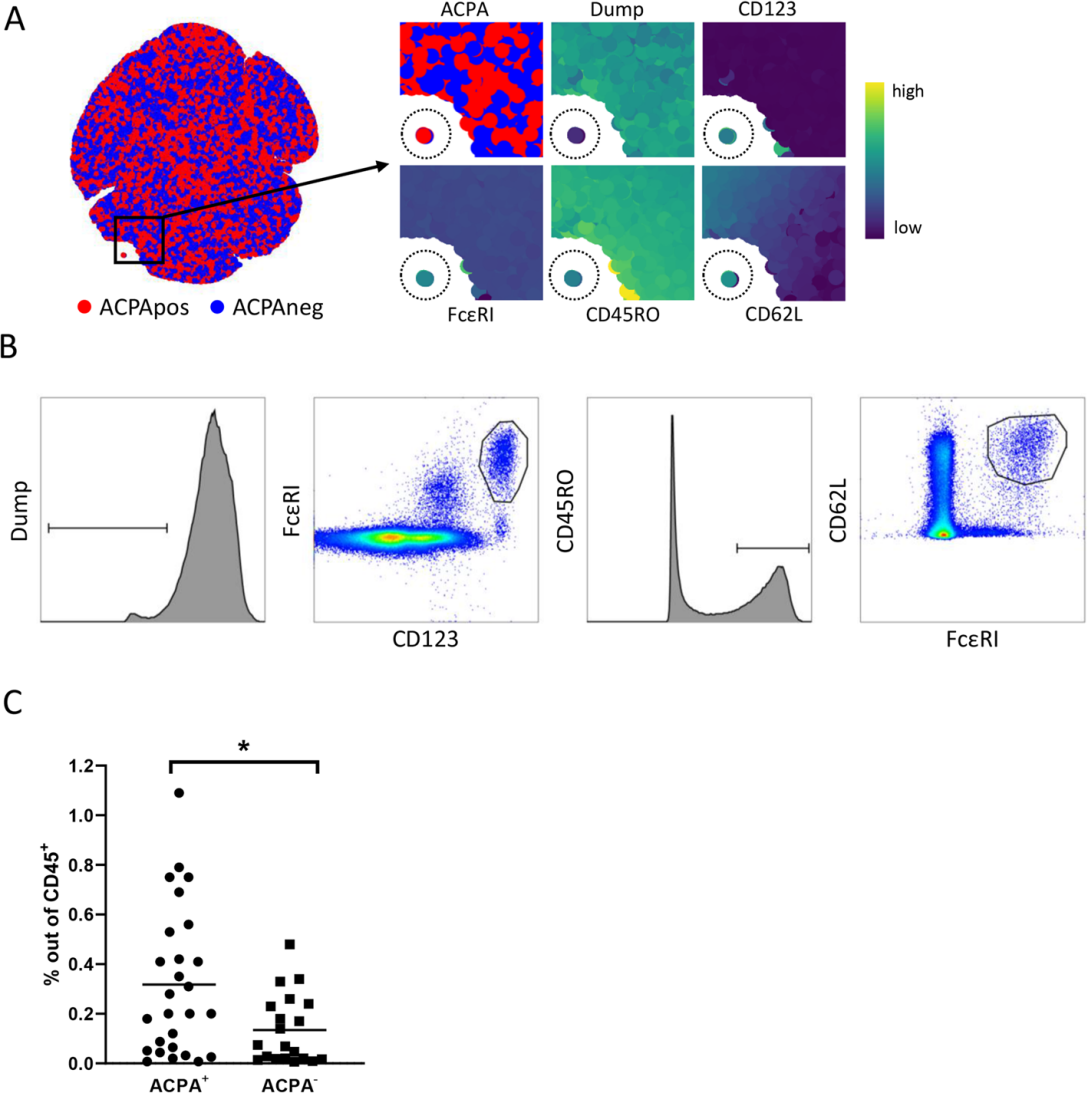
Independent flow cytometry replication confirmed the reduced presence of CD62L^+^ basophils in ACPA− samples. **A** Cytosplore dimension reduction of manually transformed flow FCS files of panel 1 showing both ACPA+ (red) and ACPA− (blue). Smaller panels on the right represent the cells within the backbox. Expression patterns matched that of MC cluster 18 (dashed circle), including CD123, FcεRI and CD45RO. CD62L expression was linked to ACPA status. **B** Gates used in Boolean gating in Flowjo to manually gate cluster 18. **C** Frequency of manually gated cells representing MC cluster 18. Subset frequency was lower in ACPA− RA (mean 0.32% vs 0.13%; *p* = 0.01)

To confirm the MC findings, we next performed a replication study using flow cytometry (FC) and an independent cohort of cryopreserved PBMCs obtained from early RA patients (*n* = 47; 27 ACPA+ vs. 20 ACPA−). All samples were split and part was used for confirmation of the basophil subset. Likewise, although the differences in NK cell subset distribution showed a clear trend but had not reached statistical significance in the discovery MC study, also the NK cell subset distribution was analysed by FC. The FC panels were designed based on the marker expression, as shown in Fig. 2A, related to either cluster 18 (panel 1) or cluster 24 (panel 2). The antibodies for FC experiments used are listed in Supplementary Table 2. FC data were pre-gated for single, live, CD45^+^ cells and exported into new FCS files, similar to MC data processing (Supplementary Figure 2). Processed files were analysed both by unsupervised analysis and manual gating. Unsupervised dimension reduction of panel 1 samples indicated a small dump-negative subset that separated from the rest of the cells (Fig. 3A). The expression pattern of this subset matched that of MC cluster 18: CD123^hi^FcεRI^+^CD45RO^med^. Intriguingly, this subset included both ACPA+ and ACPA− cells; however, only the ACPA+ cells expressed CD62L (Fig. 3A, top left and bottom right panel). These data validate our first MC findings by identifying differences between ACPA+ and ACPA− samples in the presence of a CD62L^+^ basophil-like cell population.

Based on the unsupervised FC pipeline, we determined a manual gating strategy in Flowjo for cluster 18 within the pre-gated CD45^+^ cells. To this end, four separate gates were drawn: dump negative, CD45RO positive, CD123^hi^FceRI^hi^ and CD62L positive. Boolean gating was applied to all four gates simultaneously, resulting in a subset of cells solely including cells positive for all four gates, mimicking the Cytosplore dimension reduction (Fig. 3B). Comparing ACPA+ and ACPA− patients measured by FC confirmed the MC findings as the frequency of this FC cluster was lower in ACPA− RA (mean 0.32% vs. 0.13%; *p* = 0.01, Fig. 3C). Comparing the frequency of manually gated cluster 18 to a small group of 6 healthy controls (HC) showed that HC (mean 0.32%) were more similar to ACPA+ patients and not ACPA−.

Dimension reduction of panel 2 samples resulted in a large dump-negative islet (Supplementary Figure 4). A small section of this islet partially mimicked MC cluster 24 (dashed circle), indicated by lack of CD16 and CD8 expression combined with the presence of CD45RA expression. The remainder of the markers related to cluster 24 could not clearly be identified within this section, nor was there a clear separation of cells originating from either ACPA+ or ACPA− samples. The presence of the NK subset in these cryopreserved samples therefore remains inconclusive.

## Discussion

RA patients are often separated based on their ACPA status, as the prognosis of ACPA+ is worse compared to ACPA−. Currently, a more comprehensive immune cell profile linked to ACPA status is lacking. During this study, we investigated the PBMC immune cell profile of ACPA+ and ACPA− RA at an early stage of the disease, prior to treatment initiation, using a 36-marker MC panel. Upon investigating major immune lineages, no differences were observed between ACPA+ and ACPA− patients. However, comparing early untreated ACPA+ and ACPA− RA by MC did reveal differences in two specific clusters within the innate populations. The first cluster represented a basophil-like subset expressing CD62L, CD25 and CCR6, present mostly in ACPA+ patients. The second cluster represented a CD16^−^ NK cell subset present mostly in ACPA− patients. Flow cytometry (FC) replication was performed using an independent cohort of RA patients. FC experiments confirmed the basophil MC results, showing CD62L^+^ basophils are reduced in ACPA−patients, as identified both by unsupervised analysis and manual gating. Moreover, manual gating of healthy control (HC) samples indicated that Cl-18 is indeed reduced in APCA−, as HC were more similar to ACPA+.

The NK subset identified by MC could not be confirmed by FC replication. The reason for this lack of replication is unknown. Still, it could relate to the relatively low number of patients included in the discovery cohort, the use of cells that had undergone a freeze/thaw cycle, the difference between fixed (MC) or unfixed (FC) samples, and/or differences with the patient population used for the replication studies with FC as these patients were not all naïve to therapy.

Based on previously published literature on the role of e.g. B cells and T cells in RA, we expected to find more apparent differences between these subsets when comparing major lineages. However, our MC study did not show any differences in major immune lineages. Possible explanations for this outcome could be related to the fact that a few of the MC ACPA− patients are RF+ and therefore were not completely autoantibody negative. The study size did not allow further stratification on other factors such as RF. Additionally, we included a relatively small number of patients in the ‘discovery study’ by MC analyses, and consequently, we might be underpowered to identify differences that have been reported previously. Lastly, our panel consisted of various markers to be able to investigate the overall immune cell profile of RA patients instead of running dedicated, detailed panels studying either T or B cells specifically. This could also contribute to the apparent similar distribution of T and B cell subsets in peripheral blood of ACPA− and ACPA+ patients.

Nonetheless, this study showed the value of high parameter analysis to allow broad immune cell screening of inflammatory rheumatic diseases. High-dimensional analysis reduces bias as more markers can be combined in a single experiment, resulting in the identification of unexpected cell subsets. Furthermore, our study also clearly highlighted the importance of replication: the NK cell subset observed in ACPA− disease identified by MC could not be identified by FC, indicating it is not clearly linked to disease status. This emphasizes the need for independent replication cohorts in MC studies. In contrast to the NK cell subset, the MC results for the basophil-like subset were successfully replicated by FC. The FC study included more patients than our initial MC discovery study, and therefore, these results are more accurate.

Our study utilizes PBMCs instead of whole blood, which may bias our outcome since one of the identified subsets belongs to the granulocyte lineage. Although a Ficoll-Paque gradient isolation of basophils is not uncommon, we cannot rule out that whole blood assessment of this specific CD62L+ basophil subset would have yielded different results. However, we did measure a small subset of healthy paired WB/PBMC samples and were able to identify CD62L+ basophils to a similar extend. Using PBMCs as readout may not reflect what happens in situ in the inflamed joint, but unfortunately, we were not able to obtain synovial fluid from untreated patients to further investigate this. However, a great strength of our study is the inclusion of DMARD-naïve patients in the MC study, allowing a more accurate measurement of the patient’s immune cell profile without treatment bias. Likewise, our MC study used freshly isolated PBMC samples. Certain cell types are sensitive to cryopreservation, such as e.g. plasma cells. By analysing fresh samples, there will be no (selective) cell loss due to a freeze/thaw cycle and the immune cell profile obtained is better reflecting the in situ PBMC immune cell profile. As most studies often included cryopreserved samples, this may be another reason why our study was not able to replicate reported differences between ACPA+ and ACPA− RA patients.

Cells comprising cluster 18, reduced in ACPA− samples, showed similarities with basophils based on CD123/FcƐRI/CD45RO expression, the lack of (plasmacytoid) DC markers HLA-DR/CD45RA/CD303 and absence of major lineage markers. Ficoll-Paque gradient centrifugation was performed to remove erythrocytes and granulocytes from the PBMCs; however, basophils can remain within the monolayer [20, 21]. Cytosplore analysis of MC samples indicated that cluster 18 could be separated from other CD123^+^/FcƐRI^hi^ subsets based on the expression of CD25, CD62L and CCR6. An independent FC replication supported the presence of ‘cluster 18 basophil-like cells’ identified by MC, both by dimension reduction and manual gating. Of note, the difference between ACPA+ and ACPA− was predominantly driven by CD62L expression.

The reason why CD62L+ basophil numbers are reduced in the blood of ACPA− patients as compared to ACPA+ subjects is unknown. CD62L, also known as L-selectin, is an adhesion molecule associated with early activation and rolling of leukocytes along the vessel wall. Adhesion molecules are often affected by cryopreservation resulting in decreased intensity as has been described for CD62L on T cells and CD34^+^ cells specifically [23–27]. This further emphasizes the need for the use of freshly collected material and could be a possible explanation as to why our findings have not been reported previously. Of interest, our FC cohort still indicated a difference in CD62L expression in basophils, despite the fact the samples were cryopreserved. As the expression of CD62L is very high, a decrease may not be crucial when investigating the basophils specifically in contrast to other subsets such as T cells. CD62L has been reported to be expressed by activated basophils, pointing to a possible role of these cells in the disease process [28, 29]. Although speculation, basophils could contribute to inflammatory processes in several ways. For example, data obtained in preclinical mouse models suggest a role of activated basophils in immunological memory response as depletion of basophils lowered the humoral memory response on both the T cell and B cell level, a phenomenon possibly related to the ability of basophils to bind intact antigen on their cell surface [30]. Likewise, co-culture of activated basophils with T and B cells supported B cell function and induced a ‘B helper’ phenotype in CD4^+^ T cells. In human studies, CD62L^+^ basophils have been linked to kidney diseases such as chronic kidney disease and lupus nephritis, but their contribution to disease is ill-defined [28, 29]. Considering that activated basophils could support humoral responses, it is intriguing to note that a basophil subset with an activated phenotype is less common in our ACPA− samples. It would be interesting to continue in situ studies to learn more on this subset and see if lower frequencies in the periphery correlate to increased frequencies in inflamed joints. Clearly, additional studies should be performed to investigate activated basophils in the context of autoimmunity. Moreover, it would be very interesting to investigate if the activated basophils in ACPA+ disease are actually linked to the polarization of PD-1^hi^CXCR5^−^CD4^+^ Tph cells described previously [12].

## Conclusions

Our data show a reduced population of innate cells with an activated basophil-like phenotype in ACPA− RA. The possible role of these cells in the immune response associated with RA or in other (auto)immune responses is presently unclear. However, our data provide the first evidence of a reduction of activated basophil-like cells in ACPA− disease as well as a rationale to determine their possible contribution to disease.

## Supplementary Information

**Additional file 1: Supplementary Table 1**: Antibodies used for Mass Cytometry. * in-house conjugated at 100μg scale using a Maxpar© X8 antibody labeling kit (Fluidigm)

**Additional file 2: Supplementary Table 2**: Antibodies used for Flow Cytometry. Antibodies used in FC experiments to investigate frequencies Cluster 18 (panel 1) and Cluster 24 (panel 2) identified by MC.

**Additional file 3: Supplementary Figure 1**: Gating strategy for data cleanup of Mass Cytometry data files. MC FCS files are normalized using EQ passport P13H2302 within the Fluidigm software. Gating of normalized files included sequential gating of DNA2-^193^Irridium vs Event length, followed by DNA1-^191^Irridium vs DNA2-^193^Irridium to remove doublets and debris. Cells negative for ^103^Rhodium are selected as live cells. ^140^Cerium negative cells are selected to remove EQ beads (^140^Ce^+^) followed by a selection for CD45^+^ cells. Only single/live/bead-free/CD45^+^ cells are exported into a new FCS file which was used for downstream analysis.

**Additional file 4: Supplementary Figure 2**: Gating strategy for data cleanup of Flow Cytometry files. Flow Cytometry files were unmixed within the CYTEK acquisition software based on single stained controls. Forward and Side-scatter Area are used to remove most debris. Single cells are defined through combination of Height and Area, both for Forward and Side-scatter. Live cells are defined as ZOMBIE-yellow negative. All samples are pre-gated for CD45^+^ and exported as new FCS file, similar to MC data processing.

**Additional file 5: Supplementary Figure 3**: Cytofast analysis of overview level HSNE of Mass Cytometry data. Cytosplore was used to perform dimension reduction and clustering, clusters were exported as new FCS files and loaded in R to be analyzed using the Cytofast package. A) Sample tSNE representing all MC samples. No clear pattern or separation by group was observed. B) Heatmap representing the frequency of each cluster within an MC EAC sample. The order of rows is based on the similarity of cluster-distribution within a given sample compared to other samples. C) Cytosplore analysis of all 21 clusters showing dotplots per cluster and the corresponding frequency per sample separated for ACPA. The overall distribution appears similar; however, a trend is observed for cluster 21 (T_EMRA_) present in in a few ACPA^+^ only. (ACPA+=red ACPA– =blue)

**Additional file 6: Supplementary Figure 4**: Unsupervised analysis flow replication of cluster 24 remains inconclusive. Cytosplore dimension reduction of manually transformed flow FCS files of panel 2 showing both ACPA+ (red) and ACPA– (blue). Smaller panels on the right show examples of expression per marker. Red dashed circle indicate cells thar are DUMP^–^CD16^–^CD8^–^CD45RA^+^, comparable to cluster 24. Remaining markers linked to cluster 24 (CD56, CD94, CD38 and CD161) did not clearly overlap nor did they separate based on ACPA status. Identification of cluster 24 could not be repeated by FC.

## Data Availability

The datasets used for the current study are available from the corresponding author upon reasonable request.

## Abbreviations

ACPA: Anti-citrullinated protein antibodies
ACR: American College of Rheumatology
DMARD: Disease-modifying anti-rheumatic drugs
EAC: Early Arthritis Cohort
FC: Flow cytometry
FCS: Flow cytometry standard
GMS: Gaussian mean shift
HSNE: Hierarchical stochastic neighbour embedding
ILC: Innate lymphocyte cell
MC: Mass cytometry
MFI: Mean fluorescence intensity
NK cell: Natural killer cell
PBMC: Peripheral blood mononuclear cell
RA: Rheumatoid arthritis
RF: Rheumatoid factor
tSNE: t-distributed stochastic neighbour embedding
