# Supplementary material for: Immunoprofiling of early, untreated rheumatoid arthritis using mass cytometry reveals an activated basophil subset inversely linked to ACPA status
Source: Arthritis Res Ther. 2021 Oct 29;23:272. doi: 10.1186/s13075-021-02630-8 (PMC8555233; doi:10.1186/s13075-021-02630-8)
Supplement: Supplementary file 1 — Additional file 1: Supplementary Table 1: Antibodies used for Mass Cytometry. * in-house conjugated at 100μg scale using a Maxpar© X8 antibody labeling kit (Fluidigm) [file 13075_2021_2630_MOESM1_ESM.docx]

Supplementary Table 1: Antibodies used for Mass Cytometry

| Antigen | Tag | Clone | Company | Cat# |
| --- | --- | --- | --- | --- |
| CD45 | 89Y | HI30 | FDM | 3089003B |
| CD196 | 141Pr | G034E3 | FDM | 3141003A |
| CD19 | 142Nd | HIB19 | FDM | 3142001B |
| CD117 | 143Nd | 104D2 | FDM | 3143001B |
| CD69 | 144Nd | FN50 | FDM | 3144018B |
| CD20 | 145Nd | 2H7 | BioLegend* | 302343 |
| CD8α | 146Nd | RPA-T8 | FDM | 3146001B |
| CD303 | 147Sm | 201A | FDM | 3147009B |
| CD4 | 148Nd | RPA-T4 | BioLegend* | 300541 |
| CD127 | 149Sm | A019D5 | FDM | 3149011B |
| Vα7.2 | 150Nd | 3C10 | BioLegend* | 351702 |
| CD123 | 151Eu | 6H6 | FDM | 3151001B |
| TCRγδ | 152Sm | 11F2 | FDM | 3152008B |
| CD62L | 153Eu | DREG-56 | FDM | 3153004B |
| CD3 | 154Sm | UCHT1 | FDM | 3154003B |
| CD45RA | 155Gd | HI100 | FDM | 3155011B |
| CXCR5 | 156Gd | J252D4 | BioLegend* | 356902 |
| CD27 | 158Gd | O323 | BioLegend* | 302839 |
| CD31 | 159Tb | WM59 | BioLegend* | 303127 |
| CD28 | 160Gd | CD28.2 | FDM | 3160003B |
| CD14 | 161Dy | M5E2 | BioLegend* | 301843 |
| CD11c | 162Dy | Bu15 | FDM | 3162005B |
| CD56 | 163Dy | NCAM16.2 | FDM | 3163007B |
| CD161 | 164Dy | HP-3G10 | FDM | 3164009B |
| CD45RO | 165Ho | UCHL1 | FDM | 3165011B |
| CCR7 | 167Er | G043H7 | FDM | 3167009A |
| CD199 | 168Er | L053E8 | FDM | 3168011A |
| CD25 | 169Tm | 2A3 | FDM | 3169003B |
| HLA-DR | 170Er | L243 | FDM | 3170013B |
| FcεRI | 171Yb | AER-37 | eBiosciences* | 14-5899-82 |
| CD38 | 172Yb | HIT2 | FDM | 3172007B |
| CD94 | 173Yb | HP-3D9 | BD* | 555887 |
| IL23R | 174Yb | 218213 | BioTechne* | MAB14001 |
| CD279 | 175Lu | EH12.2H7 | FDM | 3175008B |
| Ki-67 | 176Yb | B56 | BioLegend* | 350523 |
| CD16 | 209Bi | 3G8 | FDM | 3209002B |

* in-house conjugated at 100µg scale using a Maxpar^©^ X8 antibody labeling kit (Fluidigm)
