# Supplementary material for: Immunoprofiling of early, untreated rheumatoid arthritis using mass cytometry reveals an activated basophil subset inversely linked to ACPA status
Source: Arthritis Res Ther. 2021 Oct 29;23:272. doi: 10.1186/s13075-021-02630-8 (PMC8555233; doi:10.1186/s13075-021-02630-8)
Supplement: Supplementary file 2 — Additional file 2: Supplementary Table 2: Antibodies used for Flow Cytometry. Antibodies used in FC experiments to investigate frequencies Cluster 18 (panel 1) and Cluster 24 (panel 2) identified by MC. [file 13075_2021_2630_MOESM2_ESM.docx]

Supplementary Table 2: Antibodies used for Flow Cytometry

| Epitope | Fluorchrome | Clone | Company | Cat# | Panel 1 | Panel 2 |
| --- | --- | --- | --- | --- | --- | --- |
| CD3 | BV605 | UCHT1 | Biolegend | 300460 | X | X |
| CD4 | BV605 | RPA-T4 | Biolegend | 300556 | X | X |
| CD8 | BV605 | RPA-T8 | BD | 301040 | X |  |
| CD8 | BB515 | RPA-T8 | BD | 564526 |  | X |
| CD14 | BV605 | M5E2 | Biolegend | 301834 | X | X |
| CD16 | BV605 | 3G8 | Biolegend | 302040 | X |  |
| CD16 | PE-Cy7 | 3G8 | Biolegend | 302016 |  | X |
| CD19 | BV605 | HIB19 | Biolegend | 302244 | X | X |
| CD25 | APC | 2A3 | BD | 340939 | X |  |
| CD38 | BV711 | HIT2 | Biolegend | 303528 | X | X |
| CD45 | BV510 | HI30 | Biolegend | 304036 | X | X |
| CD45RA | BV605 | HI100 | Biolegend | 304134 | X |  |
| CD45RA | APC-Cy7 | HI100 | Biolegend | 304128 |  | X |
| CD45RO | APC-Cy7 | UCHL1 | Biolegend | 304228 | X |  |
| CD56 | BV786 | NCAM16.2 | BD | 564058 | X | X |
| CD62L | BB515 | DREG-1 | BD | 565037 | X |  |
| CD94 | BB700 | HP-3D9 | BD | 566534 |  | X |
| CD123 | PE-Cy7 | 6H6 | Biolegend | 306010 | X |  |
| CD127 | BV605 | A019D5 | Biolegend | 351334 |  | X |
| CD161 | AF647 | HP-3G10 | Biolegend | 339910 |  | X |
| CD196/CCR6 | BV421 | G034E3 | Biolegend | 353408 | X | X |
| CD199/CCR9 | PE | L053E8 | Biolegend | 358904 | X | X |
| FceRI | AF700 | AER-37 | Biolegend | 334630 | X |  |
| HLA-DR | BV605 | L243 | Biolegend | 307640 | X | X |
| Zombie Yellow |  |  | Biolegend | 423103 | X | X |

Antibodies used in FC experiments to investigate frequencies Cluster 18 (panel 1) and Cluster 24 (panel 2) identified by MC.
