# Supplementary figures and images for: Immunoprofiling of early, untreated rheumatoid arthritis using mass cytometry reveals an activated basophil subset inversely linked to ACPA status
Source: Arthritis Res Ther. 2021 Oct 29;23:272. doi: 10.1186/s13075-021-02630-8 (PMC8555233; doi:10.1186/s13075-021-02630-8)

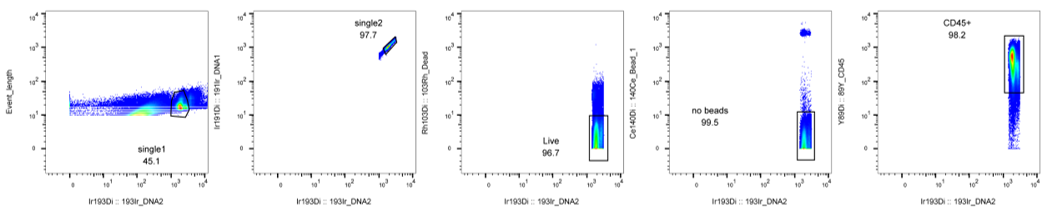

Supplement: Supplementary file 3 — Additional file 3: Supplementary Figure 1: Gating strategy for data cleanup of Mass Cytometry data files. MC FCS files are normalized using EQ passport P13H2302 within the Fluidigm software. Gating of normalized files included sequential gating of DNA2-193Irridium vs Event length, followed by DNA1-191Irridium vs DNA2-193Irridium to remove doublets and debris. Cells negative for 103Rhodium are selected as live cells. 140Cerium negative cells are selected to remove EQ beads (140Ce+) followed by a selection for CD45+ cells. Only single/live/bead-free/CD45+ cells are exported into a new FCS file which was used for downstream analysis. [file 13075_2021_2630_MOESM3_ESM.tif]

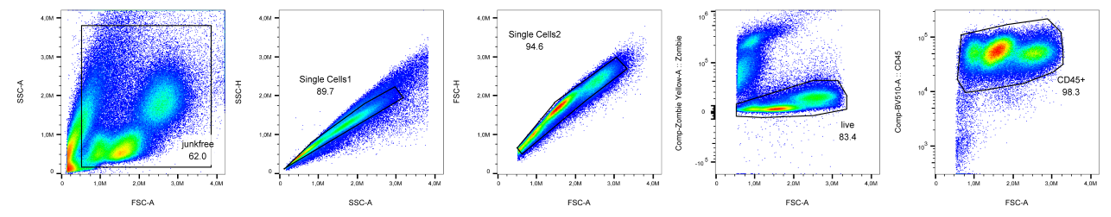

Supplement: Supplementary file 4 — Additional file 4: Supplementary Figure 2: Gating strategy for data cleanup of Flow Cytometry files. Flow Cytometry files were unmixed within the CYTEK acquisition software based on single stained controls. Forward and Side-scatter Area are used to remove most debris. Single cells are defined through combination of Height and Area, both for Forward and Side-scatter. Live cells are defined as ZOMBIE-yellow negative. All samples are pre-gated for CD45+ and exported as new FCS file, similar to MC data processing. [file 13075_2021_2630_MOESM4_ESM.tif]

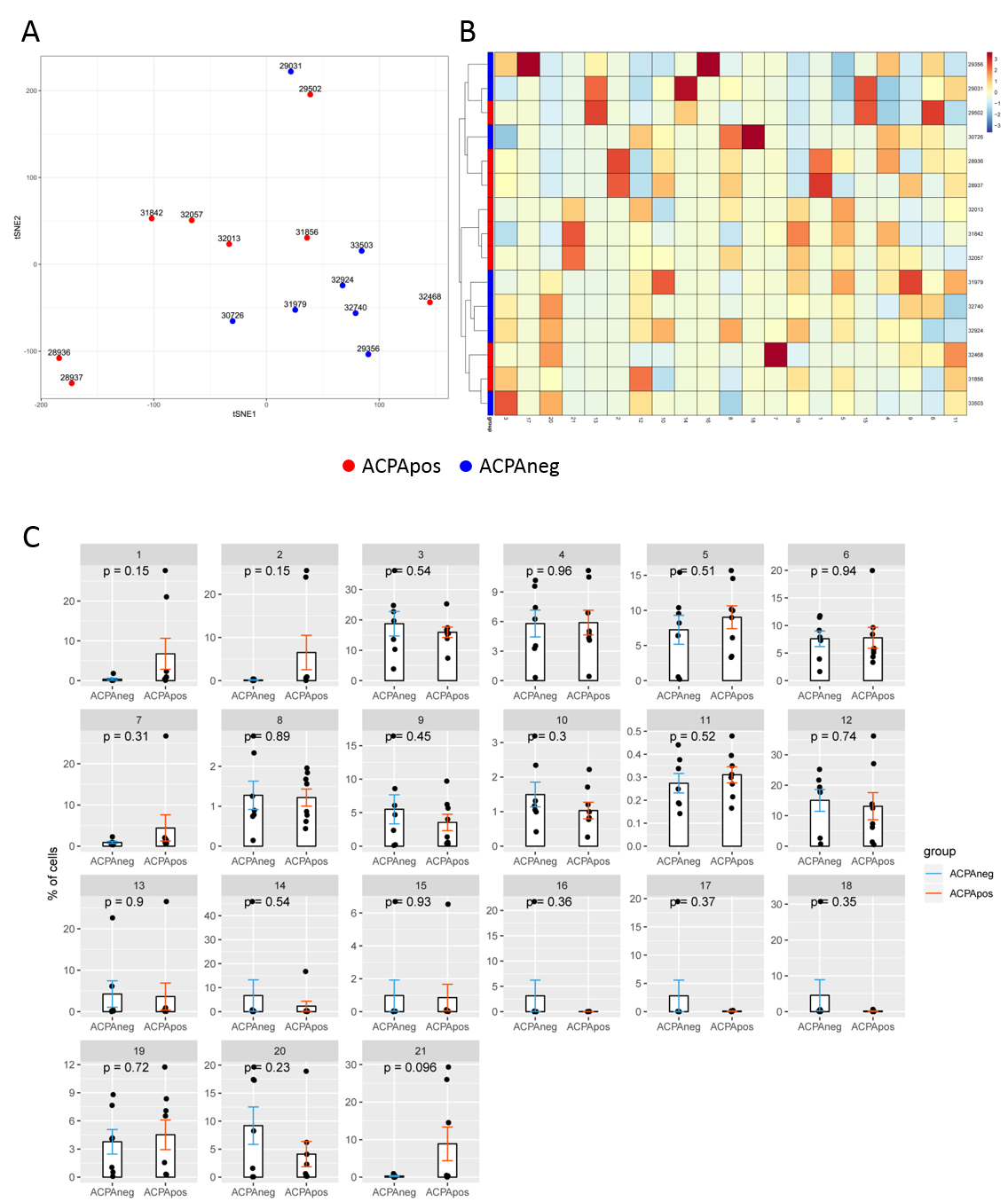

Supplement: Supplementary file 5 — Additional file 5: Supplementary Figure 3: Cytofast analysis of overview level HSNE of Mass Cytometry data. Cytosplore was used to perform dimension reduction and clustering, clusters were exported as new FCS files and loaded in R to be analyzed using the Cytofast package. A) Sample tSNE representing all MC samples. No clear pattern or separation by group was observed. B) Heatmap representing the frequency of each cluster within an MC EAC sample. The order of rows is based on the similarity of cluster-distribution within a given sample compared to other samples. C) Cytosplore analysis of all 21 clusters showing dotplots per cluster and the corresponding frequency per sample separated for ACPA. The overall distribution appears similar; however, a trend is observed for cluster 21 (TEMRA) present in in a few ACPA+ only. (ACPA+=red ACPA– =blue) [file 13075_2021_2630_MOESM5_ESM.tif]

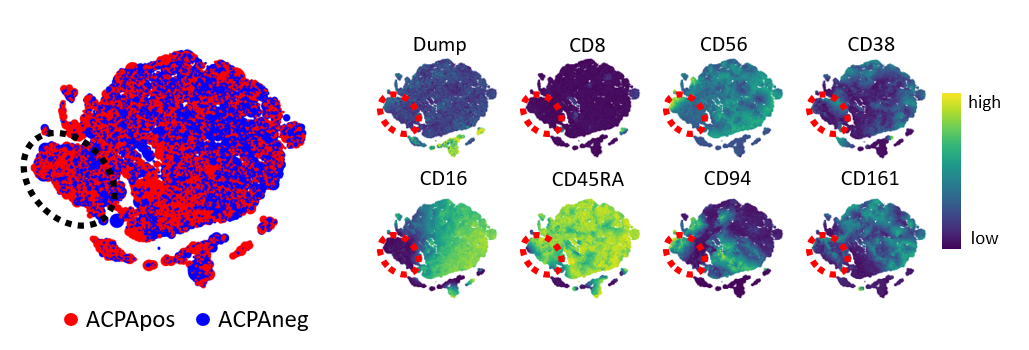

Supplement: Supplementary file 6 — Additional file 6: Supplementary Figure 4: Unsupervised analysis flow replication of cluster 24 remains inconclusive. Cytosplore dimension reduction of manually transformed flow FCS files of panel 2 showing both ACPA+ (red) and ACPA– (blue). Smaller panels on the right show examples of expression per marker. Red dashed circle indicate cells thar are DUMP–CD16–CD8–CD45RA+, comparable to cluster 24. Remaining markers linked to cluster 24 (CD56, CD94, CD38 and CD161) did not clearly overlap nor did they separate based on ACPA status. Identification of cluster 24 could not be repeated by FC. [file 13075_2021_2630_MOESM6_ESM.tif]
